# Positive schizotypy is associated with amplified mnemonic discrimination and attenuated generalization

**DOI:** 10.1007/s00406-022-01430-8

**Published:** 2022-05-27

**Authors:** Ágota Vass, Melinda Becske, Ágnes Szőllősi, Mihály Racsmány, Bertalan Polner

**Affiliations:** 1 Department of Cognitive Science, Budapest University of Technology and Economics, Egry József u. 1., Budapest, 1111 Hungary; 2 Department of Psychiatry and Psychotherapy, Semmelweis University, Balassa u. 6., Budapest, 1083 Hungary; 3 Institute of Cognitive Neuroscience and Psychology, Research Centre for Natural Sciences, Magyar tudósok körútja 2., Budapest, 1117 Hungary

**Keywords:** Hippocampus, Memory, Odd beliefs, Pattern completion, Pattern separation

## Abstract

Tendency to experience inaccurate beliefs alongside perceptual anomalies constitutes positive schizotypal traits in the general population and shows continuity with the positive symptoms of schizophrenia. It has been hypothesized that the positive symptomatology of schizophrenia, and by extension, the odd beliefs and unusual perceptual experiences in the general population, are associated with specific alterations in memory functions. An imbalance between memory generalization and episodic memory specificity has been proposed on several counts; however, the direction of the imbalance is currently unclear. Here, we evaluated the association between positive schizotypy, and memory alterations related to hippocampal computations in a general population sample enriched for positive schizotypy. We found that memory generalization is attenuated while memory specificity is elevated in participants with more pronounced positive schizotypal traits. Our findings show that people who are prone to irrational beliefs and unusual experiences also show measurable alterations in memory and likely have difficulty grasping the global picture and rather be overpowered by fragments of information.

**Supplementary Information:**

The online version contains supplementary material available at 10.1007/s00406-022-01430-8.

## Introduction

Schizotypal personality traits have been conceptualized to form a continuum with symptoms of schizophrenia [1] which is evidenced by marked phenomenological and cognitive overlaps [1, 2] and higher probability of transition into psychosis in people with more pronounced schizotypal traits [3]. While schizophrenia is a severe neuropsychiatric disorder with a prevalence rate of under 1% [4], schizotypal traits represent a mild analogue of the symptoms of schizophrenia and form part of the natural variation of personality traits in the general population [1, 3, 5]. Schizotypy comprises multiple dimensions, one of which is positive schizotypy, that implies proneness to odd, delusion-like beliefs, and unusual, hallucination-like experiences [1]. Such tendencies correlate with Big Five personality traits such as higher Neuroticism and Openness [6, 7].

It has been suggested that the positive symptoms of schizophrenia are related to specific learning and memory alterations [8] implying that people who are prone to irrational beliefs and unusual perceptions may also demonstrate distinct alterations in memory functions. There is a discrepancy in the literature, however, regarding how positive symptoms are related to such alterations in memory. According to Tamminga and colleagues, the positive symptomatology of schizophrenia is linked to the imbalance of hippocampal neural computations [9, 10]. Specifically, in their’hippocampal homeostatic plasticity model of psychosis in schizophrenia’, Tamminga and colleagues [10] argued convincingly that given the hippocampus malformations that can be detected in schizophrenia, specific relationships can be hypothesized between pattern separation and pattern completion functions and psychosis. Namely, altered, inaccurate and biased pattern separation and pattern completion functions could generate psychotic content, especially delusions and thought disorder.

The hippocampus is a medial temporal lobe structure that is associated with episodic memory [11] and is assumed to play a leading role in memory specificity and generalization [12]. Memories are likely to overlap with each other in content which hinders the retrieval of unique memories. Storing specific details in the face of significant overlap between memory representations is carried out by pattern separation, a neural computation in the hippocampus. As a result of pattern separation, the neuronal activities of brain circuits become distinct for two or more stimuli that share similar features [13, 14]. On the other hand, the unification of related memory elements into an integrated unit is attributed to the neural computation of pattern completion. Pattern completion relies on an autoassociative network in the hippocampus that fills in incomplete incoming information based on previously stored representations [13] and refers to the process when a memory is accessed in response to partial or degraded cues [15–17]. Since memory generalization allows the extraction of regularities from discrete but similar experiences, it seems plausible that it is supported by pattern completion at the computational level [12, 18].

While it is assumed that the imbalance between neural computations related to episodic memory is associated with positive symptoms, the direction of the imbalance is unclear. On the one hand, increased memory generalization and deteriorated specificity were assumed to share a common ground with hallucinations and delusions [9, 10]. In terms of underlying neural mechanisms, this translates into amplified pattern completion and attenuated pattern separation, as pattern completion is assumed to be associated with memory generalization and pattern separation is assumed to be related to memory specificity [12]. The findings of Das and colleagues [19], Martinelli and Shergill [20], as well as Kraguljac and colleagues [21] supported this framework of a bias towards reduced memory specificity in schizophrenia. All studies found the behavioral manifestation of pattern separation to be weaker in schizophrenia, however, no relationship between positive symptoms and memory biases was revealed. The results are likely to be confounded by relatively small samples, the effect of antipsychotic medications [22] and may reflect a generalized performance deficit rather than a function-specific alteration [23].

While these studies were motivated by the hypothesis [9, 10] that a bias towards reduced memory specificity may be related to the positive symptoms of schizophrenia, the long-standing literature of the disorder that dates as far back as to the 1900’s suggests otherwise [24]. Schizophrenia has been described as a disorder of fragmentation with a distorted sense of subjectivity [25] and by the loss of sense of continuity both to one’s self and perceptual organization [26]. Relatedly, studies specifically assessing memory alterations have found that positive symptoms correlated with an increased tendency to remember the local as opposed to the global features of the stimuli [27, 28]. Such alterations are rather translatable into amplified memory specificity, implying overactive pattern separation and a bias towards reduced generalization, which would correspond to attenuated pattern completion at the neural level; at the phenomenological level, these would be reflected in a sense of fragmentation, a central feature of psychosis [29].

### Motivation and aims

It is currently unclear how memory formation may be altered in relation to the positive symptoms of schizophrenia and, by extension, in people who are prone to irrational or delusion-like belief formation and unusual perceptual experiences. In particular, two opposing hypotheses emerged from the literature. On the one hand, the work of Tamminga and colleagues [9, 10], as well as empirical studies examining clinical samples [19–21] supported the hypothesis that there is a bias towards reduced memory specificity and enhanced generalization in schizophrenia. On the other hand, countless accounts in the long-standing literature on memory alterations in schizophrenia [27, 28, 30, 31] and general phenomenology of the disorder [24, 25] argue that it is rather the process of generalization that is defective, and the disorder leaves people incapacitated to integrate fragments into a whole, implying amplified specificity.

Here, for the first time, we aimed to contrast these two competing hypotheses, which predict opposing associations of positive schizotypy with memory specificity and generalization. Thus, we were able to simultaneously evaluate the two hypotheses within the same statistical models. To avoid confounding by illness-related factors such as hospitalization and medication, we recruited a sample from the general population. To achieve higher statistical power, we increased variability in the sample by oversampling for positive schizotypy. We assessed memory specificity and generalization, as behavioral indicators of pattern separation and completion, respectively, with a modified recognition memory task, the well-established Mnemonic Similarity Task [MST; 32]. We fitted linear regression models to evaluate the relationship between positive schizotypy and memory specificity vs. generalization. We established the high reliability of our key measurements. Robustness of the associations were tested by adjusting the models for age and gender, and by additional control analyses adjusting for perceptual deficits, negative, disorganized, and impulsive dimensions of schizotypy, and additional aspects of psychopathology.

## Materials and methods

### Participants

We have oversampled for high positive schizotypy to ensure sufficient variability, as our aim was to specifically focus on the positive dimension. As part of the oversampling, 614 people filled out an online form that included the positive and negative schizotypy subscales from the short Oxford-Liverpool Inventory of Feelings and Experiences Questionnaire (O-LIFE; for details, see below) and a question about the age of the respondent. The form was posted in Facebook groups of students studying at various universities based in Budapest. The form selected participants based on the following criteria: age between 18 and 35 years, achieve 7 out of 12 points or higher on the Unusual Experiences subscale that assesses positive schizotypy, and 3 out of 12 points or lower on the Introvertive Anhedonia subscale that assesses negative schizotypy (these cut-offs were based on previous large university student samples [e.g. 33, 34].

One hundred ninety-one people met the criteria and 23 of them volunteered to participate in the study. In addition, a convenience sample of 64 participants were recruited through university courses and social media advertisements. Some of the participants received course credit as a form of compensation. Further, 11 out of the total of 87 participants were excluded from the final analysis due to a self-reported history of neurological or psychiatric disorders, epilepsy, or traumatic brain injury. Five participants were excluded because they were identified by Cook’s Distance as influential points distorting the results of our regression models (see details in “Results”). Thus, the final sample comprised 71 participants (see Table 1 for descriptives).^1^

**Table 1:** Descriptive statistics of the sample

	Convenience student sample (*N* = 53)	High positive schizotypy student sample (*N* = 18)	Total (*N* = 71)
Sex			
Female	41 (77.4%)	13 (72.2%)	54 (76.1%)
Male	12 (22.6%)	5 (27.8%)	17 (23.9%)
Age (years)			
Mean (SD), [Min, Max]	24.7 (5.6), [19.0, 49.0]	22.2 (2.9), [18.0, 27.0]	24.0 (5.1), [18.0, 49.0]
Education (years)			
Mean (SD), [Min, Max]	16.3 (2.21), [12.0, 21.0]	15.5 (2.62), [12.0, 20.0]	16.1 (2.33), [12.0, 21.0]
Lure discrimination index			
Mean (SD), [Min, Max]	0.35 (0.17), [0.05, 0.69]	0.37 (0.14), [0.09, 0.55]	0.36 (0.16), [0.05, 0.69]
False recognition of lures			
Mean (SD), [Min, Max]	0.39 (0.13), [0.19, 0.63]	0.31 (0.10), [0.14, 0.47]	0.37 (0.13), [0.14, 0.63]
Positive schizotypy			
Mean (SD), [Min, Max]	3.4 (2.6), [0, 10.0]	7.1 (2.0), [3.0, 10.0]	4.3 (2.9), [0, 10.0]

Participants provided written informed consent. The authors assert that all procedures contributing to this work comply with the ethical standards of the relevant national and institutional committees on human experimentation and with the Helsinki Declaration of 1975, as revised in 2008. The study was approved by the United Ethical Review Committee for Research in Psychology, Hungary (2016/032).

### Questionnaires

Participants completed several self-administered psychometric instruments to quantify their level of schizotypy and general mental well-being from a variety of perspectives. Overall, the instruments had good reliability in the sample (*α* >  = 0.7, except for negative and impulsive schizotypy; see details in Supplementary Materials and distributions in Supplementary Fig. 1). Participants completed the short version of the O-LIFE questionnaire [38] [Hungarian version:, 39] which measures schizotypal personality traits. As sleep disturbances have been shown to be correlated with schizotypy [40], participants completed the Athens Insomnia Scale (AIS) [41] [Hungarian version:, 42]. To obtain information on psychopathological states, participants also filled questionnaires about momentary psychotic-like experiences [43] and state anxiety [44] [Hungarian version:, 45]. Higher scores in all questionnaires correspond to an indicator of pathological functioning, except for the General Health Questionnaire-12 (GHQ-12) [46] [Hungarian version:, 47], where a higher score corresponds to better mental health.

### Mnemonic similarity task

**Fig. 1:**
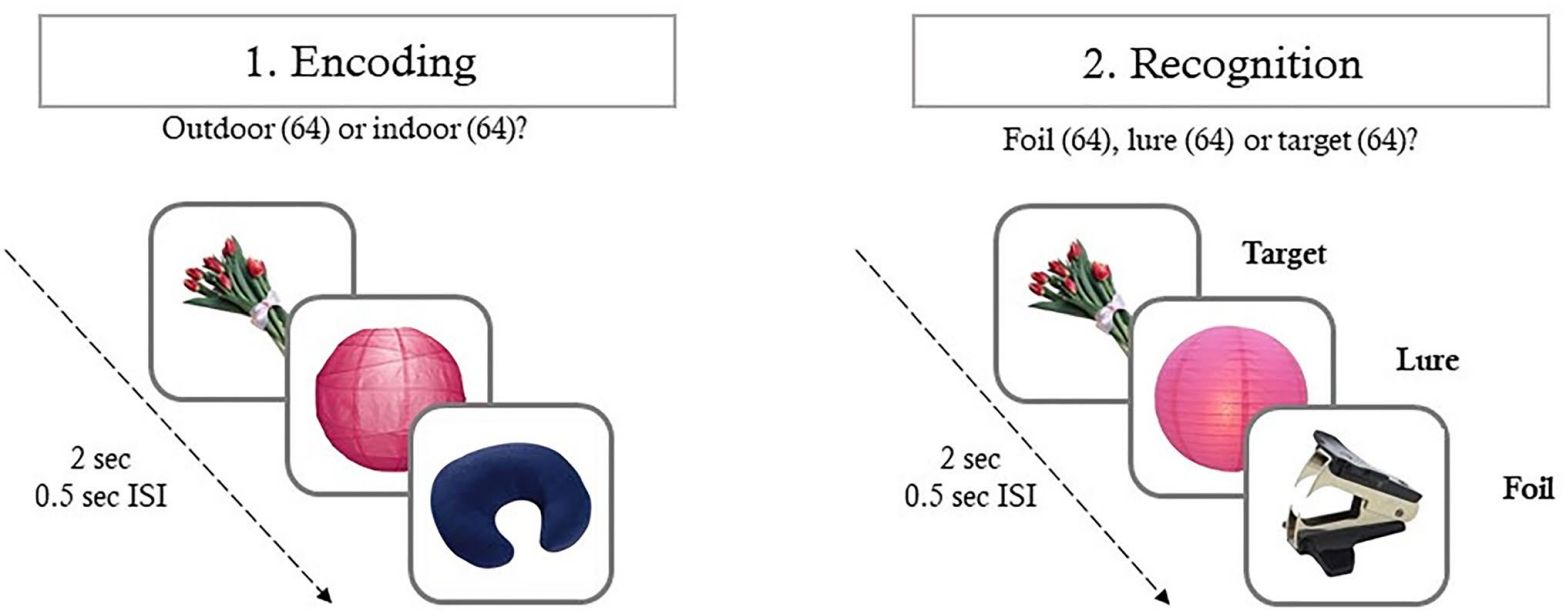
The design and the experimental procedure of the Mnemonic Similarity Task. Participants were presented 128 pictures of everyday objects in the encoding phase. In the recognition phase, participants were shown 192 pictures of everyday objects, 64 of which were exact repetitions of objects shown in the encoding phase (targets), 64 were new objects (foils) and 64 were perceptually similar items to ones presented in the encoding phase of the task (lures). *ISI* = inter-stimulus interval

The task is illustrated in Fig. 1. In the incidental encoding phase of the task, participants made simple decisions about pictures of everyday objects. Unbeknownst to participants, this was followed by a recognition phase, where participants were asked to make memory judgements about pictures of everyday objects that were either identical, visually similar, or completely different from the pictures shown in the encoding phase (targets, lures, and foils, respectively). The target and lure images were visually similar. Similarity was manipulated between the targets and lures across a wide range of characteristics, for example the color, shape, and size of the objects. Following previous studies [see 32], the discrimination performance between the studied old items and their visually similar lure pictures (the ratio of “similar” responses given to the lure items) was used as an indicator of memory specificity (widely known as the so-called Lure Discrimination Index; LDI), while false recognition of similar lure pictures (the ratio of “old” responses given to the lure items) was used as an index of memory generalization. We note that the LDI and false recognition of the lures in the MST are widely used behavioral measures of pattern separation and completion, respectively [14, 48]. Both indices had good split-half reliability in the sample (0.80 and 0.78, respectively). For the detailed description of the task, see Supplementary Materials and [32].

### Perceptual discrimination task

To control for perceptual deficits, participants took part in a perceptual discrimination (PD) test immediately after the completion of the MST. Participants judged whether 90 pairs of images were identical to, different from, or similar to each other. Stimuli were colorful images of objects. Each trial consisted of the subsequent presentations of two images and participants were asked to identify them correctly as “old”, “new”, or “similar” via button press. For the detailed description of the task, see Supplementary Materials.

## Results

We have carried out a series of linear regression analyses to examine the relationship between behavioral indicators of pattern separation (lure discrimination index; LDI) and pattern completion (false recognition of the lure stimuli) and positive schizotypy scores. The assumptions of homoscedasticity and normally distributed residuals had been tested and met. Since data points with large residuals or high leverage may distort the outcome and accuracy of a regression model, we have excluded five participants based on Cook’s distance. Age and sex were included in all models. The rationale for entering age as a control variable was that discrimination performance is known to be affected in the elderly [32, 48]. To assess the specificity of the relationships between positive schizotypy and the LDI as well as the false recognition of lures, we have carried out control analyses, where the model was extended with one of the following variables: perceptual discrimination performance; negative, disorganized, or impulsive schizotypy; insomnia; general mental health; momentary psychotic-like experiences; state anxiety. The results are summarized in Table 2.

**Table 2:** Summary of linear regression models predicting lure discrimination/false recognition of lures from positive schizotypy, before and after adjustment for control variables

	Covariate: -	Covariate: perceptual discrimination	Covariate: negative schizotypy	Covariate: disorganized schizotypy	Covariate: impulsive schizotypy	Covariate: insomnia	Covariate: General mental health	Covariate: psychotic experiences	Covariate: state anxiety
Positive schizotypy	0.015* [0.003, 0.028]	0.014* [0.001, 0.027]	0.015* [0.002, 0.028]	0.015* [0.001, 0.028]	0.015* [0.001, 0.029]	0.015* [0.003, 0.028]	0.015* [0.003, 0.028]	0.019** [0.006, 0.033]	0.015* [0.003, 0.028]
Covariate	NA	0.094 [− 0.181, 0.369]	− 0.006 [− 0.035, 0.023]	0.002 [− 0.013, 0.018]	0.001 [− 0.024, 0.026]	0.001 [− 0.01, 0.013]	− 0.001 [− 0.008, 0.007]	− 0.006 + [− 0.013, 0.001]	− 0.002 [− 0.007, 0.002]
Age	− 0.006 + [− 0.013, 0.001]	− 0.006 + [− 0.014, 0.001]	− 0.006 [− 0.013, 0.001]	− 0.006 + [− 0.013, 0.001]	− 0.006 [− 0.014, 0.002]	− 0.006 [− 0.013, 0.001]	− 0.006 + [− 0.013, 0.001]	− 0.007 + [− 0.014, 0]	− 0.007 + [− 0.014, 0]
Sex (female)	− 0.01 [− 0.094, 0.075]	− 0.016 [− 0.103, 0.071]	− 0.01 [− 0.096, 0.075]	− 0.011 [− 0.097, 0.075]	− 0.009 [− 0.095, 0.077]	− 0.011 [− 0.097, 0.075]	− 0.01 [− 0.096, 0.076]	− 0.004 [− 0.088, 0.08]	− 0.006 [− 0.091, 0.079]
*F*	3.57*	2.77*	2.69*	2.67*	2.64*	2.65*	2.64*	3.45*	2.95*
*df*	3,67	4,66	4,66	4,66	4,66	4,66	4,66	4,66	4,66
*R*^2^	0.14	0.14	0.14	0.14	0.14	0.14	0.14	0.17	0.15
adj.*R*^2^	0.1	0.09	0.09	0.09	0.09	0.09	0.09	0.12	0.1

	Covariate: -	Covariate: perceptual discrimination	Covariate: negative schizotypy	Covariate: disorganized schizotypy	Covariate: impulsive schizotypy	Covariate: insomnia	Covariate: General mental health	Covariate: psychotic experiences	Covariate: state anxiety
Positive schizotypy	− 0.016** [− 0.026, − 0.007]	− 0.017** [− 0.027, − 0.006]	− 0.015** [− 0.025, − 0.005]	− 0.018** [− 0.028, − 0.008]	− 0.016** [− 0.026, − 0.005]	− 0.016** [− 0.026, − 0.007]	− 0.016** [− 0.026, − 0.007]	− 0.019*** [− 0.029, − 0.009]	− 0.016** [− 0.026, − 0.007]
Covariate	NA	− 0.054 [− 0.573, 0.465]	0.015 [− 0.007, 0.036]	0.005 [− 0.006, 0.017]	− 0.003 [− 0.022, 0.016]	− 0.001 [− 0.01, 0.007]	0.001 [− 0.005, 0.006]	0.004 + [− 0.001, 0.01]	0.002 [− 0.002, 0.005]
Age	0.007* [0.001, 0.012]	0.007* [0.001, 0.012]	0.006* [0.001, 0.012]	0.007* [0.001, 0.012]	0.006* [0.001, 0.012]	0.007* [0.001, 0.012]	0.007* [0.001, 0.012]	0.007** [0.002, 0.013]	0.007* [0.002, 0.013]
Sex (female)	0.02 [− 0.045, 0.085]	0.02 [− 0.045, 0.086]	0.021 [− 0.043, 0.086]	0.017 [− 0.048, 0.082]	0.019 [− 0.046, 0.084]	0.021 [− 0.045, 0.087]	0.02 [− 0.045, 0.086]	0.016 [− 0.048, 0.08]	0.017 [− 0.048, 0.082]
*F*	7.07***	5.24**	5.83***	5.48***	5.26***	5.25***	5.23**	6.2***	5.55***
*df*	3,67	4,66	4,66	4,66	4,66	4,66	4,66	4,66	4,66
*R*^2^	0.24	0.24	0.26	0.25	0.24	0.24	0.24	0.27	0.25
adj.*R*^2^	0.21	0.19	0.22	0.2	0.2	0.2	0.19	0.23	0.21

Dependent variable: LDI. Dependent variable: false recognition of lures

*LDI* lure discrimination index. When adjusting for performance on the perceptual discrimination (PD) task, the LDI on the PD was added when predicting the LDI on the MST, and false recognition of lures on the PD task was added when predicting false recognition of lures on the MST. For each predictor, unstandardized coefficient estimates and their 95% confidence intervals are shown, rounded to 3 digits. Model fit statistics are rounded to 2 digits

*N* = 71, ***p < 0.001, **p < 0.01, *p < 0.05, ^+^*p* < 0.1

### Lure discrimination/false recognition of lures and positive schizotypy

**Fig. 2:**
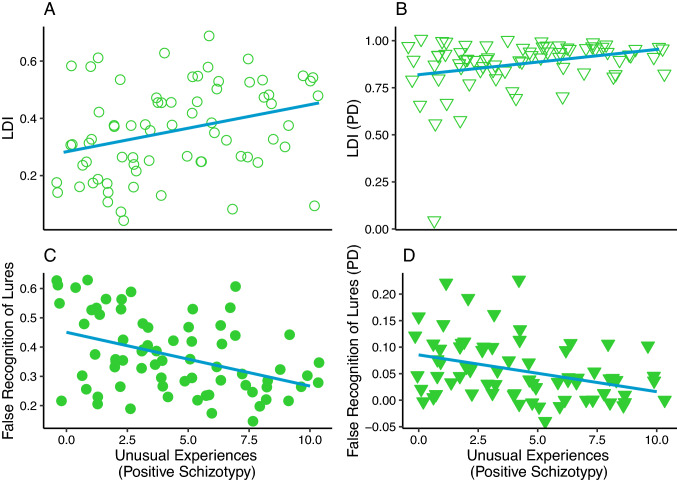
The association between positive schizotypy (O-LIFE) and lure discrimination/false recognition of lures achieved in the MST and PD tests. *Note(s).* The *x*-axis shows positive schizotypy and the *y*-axis shows the MST (‘**A**’ and ‘**C**’) and PD performance scores (‘**B**’ and ‘**D**’). Linear trendlines are shown

**Fig. 3:**
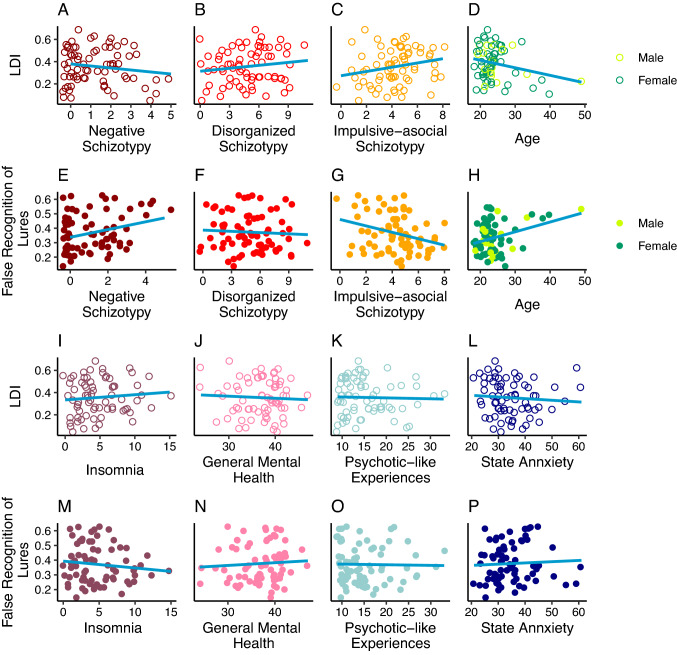
The association between negative, disorganized and impulsive-asocial dimensions of schizotypy, age and gender, further trait- and state-like psychopathology and lure discrimination/false recognition of lures achieved in the MST. The *x*-axis shows the different schizotypy dimensions (‘**A**’–‘**C**’, ‘**E**’–‘**G**’), age and gender (‘**D**’, ‘**H**’), and further trait- and state-like psychopathology (‘**I**’–‘**P**’). The *y*-axis shows the MST performance scores. Linear trendlines are shown

Firstly, we investigated the relationship between the LDI as well as false recognition of lures and positive schizotypy. If a higher score on the unusual experiences scale of the O-LIFE questionnaire is to be associated with an attenuated LDI and a higher number of false recognition of lures, then our results would lend support to the hypothesis of a bias towards reduced memory specificity and enhanced generalization. However, if higher positive schizotypy scores are to be associated with an amplified LDI and lower false recognition of lures, then our results would be in line with the hypothesis of a bias towards reduced memory generalization and enhanced specificity. Critically, we found that positive schizotypy was significantly related to enhanced lure discrimination and lower false recognition of lures (see Table 2, first column and Figs. 2A, C). This effect was significant over and above the effect of age and gender. However, we should also note that age showed significant relationships with task performance. Attenuated mnemonic discrimination performance and enhanced false recognition of lures were more likely to be observed in relatively older participants in our sample, although the former effect was marginally significant (also see Figs. 3D, H). In the following analyses, we evaluated the robustness and specificity of these associations.

### Control analyses: perceptual discrimination

First, we considered the possibility that deficits in perception and early visual information processing may cascade to later stages of information processing. Thus, we have examined whether the significant effects detected could be explained by elementary perceptual deficits [20]. For this purpose, we have estimated the LDI and false recognition of lures on the PD test for each individual. Our results showed a significant relationship between performance on the PD test and the O-LIFE Unusual Experiences score (see Fig. 2B, D). Higher positive schizotypy was associated with enhanced lure discrimination performance (β(SE) = 0.014 (0.006), *p* = 0.01, *F*(3,67) = 3.08. *p* = 0.03, Adjusted *R*^2^ = 0.08) and attenuated false recognitions of lures (β(SE) = − 0.007 (0.002), *p* = 0.01, *F*(3,67) = 3.58, *p* = 0.02, Adjusted *R*^2^ = 0.10) on the PD test, over and above the effect of age (LDI on the PD: β(SE) = 0.001(0.003), *p* = 0.79; false recognition of lures on the PD test: β(SE) = 0.01(0.001), *p* = 0.35) and sex (LDI on the PD: β(SE) = 0.67(0.038), *p* = 0.08; false recognition of lures on the PD test: β(SE) = 0.009(0.015), *p* = 0.57). However, discrimination and false recognition of lures on the MST were not significantly associated with PD test performance, and the effect of positive schizotypy on performance on the MST remained significant after adjusting for the non-significant effects of PD indices (see Table 2, second column). Overall, these findings suggest that although positive schizotypy is associated with altered perceptual discrimination performance, this does not explain the observed alterations in memory specificity and generalization.

### Control analyses: negative, disorganized and impulsive-asocial dimensions of schizotypy

We considered three additional dimensions of schizotypy beyond positive schizotypy and examined whether the relationship between putative behavioral indicators of hippocampal neural computations and positive schizotypy is specific, or, whether it can be extended to the negative, disorganized, and impulsive-asocial dimensions of schizotypy. None of these O-LIFE dimensions had a statistically significant relationship with LDI when added as second explanatory variables (see Table 2, third to fifth column and Fig. 3A–C and Fig. 3E–G). After adjusting for these non-significant effects, the associations between positive schizotypy and lure discrimination as well as the false recognition of lures did not change essentially, providing evidence for their robustness.

### Control analyses: further trait- and state-like psychopathology

To further test for the robustness of the effect of positive schizotypy, we have constructed regression models where we added insomnia, general mental health, momentary psychotic-like experiences, and state anxiety, respectively, as additional explanatory variables alongside positive schizotypy. Our results showed that none of them had a statistically significant effect on the LDI or the false recognition of lures, and the effect of positive schizotypy was robust (see Table 2, last four columns and Fig. 3I–P).

### Control analyses: overall recognition memory

Finally, to further evaluate the specificity of the findings, we assessed the relationship of positive schizotypy and overall recognition memory performance (‘old’ responses to targets minus ‘old’ responses to foils). Positive schizotypy was not significantly associated with recognition memory performance (β(SE) = 0.001 (0.005), *p* = 0.79, *F*(3,67) = 0.42, *p* = 0.74, Adjusted *R*^2^ = − 0.03), over and above the non-significant effects of age (β(SE) = 0.003 (0.003), *p* = 0.27) and sex (β(SE) =—0.001 (0.031), *p* = 0.97). This result is consistent with the conjecture that positive schizotypy is associated with a specific alteration of memory and not a general impairment of recognition.

## Discussion

Positive schizotypy in the general population refers to a tendency of experiencing perceptual aberrations and odd beliefs, and it seems to form a continuum with the positive symptoms of schizophrenia such as hallucinations and delusions [1]. It has been established that these symptoms are associated with specific memory alterations, however, two opposing hypotheses exist that outline the nature of these alterations. Our aims in the current study were twofold. Firstly, as there is a considerable phenomenological and cognitive overlap between schizophrenia and schizotypy [1], we tested whether people with elevated positive schizotypal traits also demonstrate specific alterations in memory. Importantly, by recruiting individuals from the general population, we minimized confounding by illness-related factors such as medication or hospitalization, which was a potential limitation of previous patient studies. Secondly, we evaluated two contrasting hypotheses and tested whether people with more pronounced positive schizotypal traits were more prone to overgeneralize when remembering or memorizing rather the local than the global features of events.

Specifically, in line with previous work studying memory generalization and specificity at the behavioral level, we adapted the MST [32] to measure the behavioral manifestation of specific hippocampal neural computations. The LDI corresponded to the behavioral proxy of pattern separation and provided an estimate of the ability to distinguish perceptually similar stimuli, thus, to discriminate between overlapping memory representations. False recognition of lures corresponded to the behavioral proxy of pattern completion and served as an estimate of the capability to generalize across perceptually similar features. In our study, we used the original version of the Mnemonic Discrimination Task [48] which includes three (and not two) response options:’old’,’new’, and’similar’. Due to this aspect of the task, the ratio of’similar’ responses to lures and the ratio of’old’ responses to the lures are not complementary to each other. Accordingly, the processes of pattern separation and pattern completion are suggested to be associated with different aspects of episodic memory (mnemonic discrimination via interference resolution and holistic recollection, respectively; [13]). We note, however, that the two behavioral indices are interrelated (LDI and False Recognition of Lures were significantly correlated in our sample: *r* = 0.71, *p* < 0.001, corresponding to ~ 50% shared variance). In fact, the hippocampal computations of pattern separation and pattern completion and also their behavioral manifestations (lure discrimination and generalization, respectively) always work closely together (e.g. [48, 49]). Whenever one encounters a stimulus that is similar to (but not the same as) a previously perceived stimulus, one may demonstrate a bias either toward correct discrimination or toward generalization, but not toward both. According to our results, a bias toward reduced false recognition (and consequently, to attenuated generalization) is present in individuals with more pronounced positive schizotypal traits. In other words, these individuals are less likely to access prototypical category exemplars in a memory task when there is an overlap between the studied and test stimuli. Instead, they tend to access specific items together with their distinguishable properties. In sum, it never happens that one has a tendency toward pattern separation and completion at the same time. Over and above the effects of age and gender, perceptual discrimination, other dimensions of schizotypy and associated psychopathology, positive schizotypy was consistently associated with enhanced memory specificity and attenuated generalization. Thus, our results support the hypothesis that has arisen from the long-established literature describing schizophrenia as a disorder of fragmentation at several levels [24, 29].

### Bias towards reduced generalization

We found that the more pronounced positive schizotypal traits an individual possesses, the more they tend to demonstrate a bias towards reduced mnemonic generalization. This is in line with evidence suggesting that generalization processes are compromised in schizophrenia [22, 50]. Attenuated pattern completion can be conceptualized as one possible mechanism underlying lower generalization performance [12], as they both involve the extraction of common regularities from discrete but similar experiences [12, 18].

According to influential models of autobiographical memory, episodic memories are formed by a balanced interaction of encoded experience-near record of ongoing activity and the simultaneous need to maintain a coherent and stable record of the self’s interaction with the world that extends beyond the present moment [51, 52]. Extracting commonalities regarding one’s self from different experiences which are robust and permanent presumably requires the ability to generalize. Relatedly, it has been argued that a cohesive self develops out of an interaction between numerous ‘selves’ that are experienced through a wide variety of different situations [53]. In line with the theory of weaker generalization processes, people diagnosed with schizophrenia were also found to have a decreased self-concept clarity [53]. This implies that patients show a bias towards reduced generalization of common features regarding the self from various events. Specifically, self-concept clarity was negatively associated with positive symptoms of schizophrenia. Additionally, attenuated self-concept clarity has also been found to co-occur with the positive, negative, and disorganized factors of schizotypy [54, 55].

Taken together, the tendency to generalize less across several domains in schizophrenia and schizotypy strongly support our findings that the behavioral proxy of pattern completion is negatively linked to the positive dimension of schizotypy, implying that higher positive schizotypal traits are associated with a bias towards reduced generalization.

### Amplified specificity

Higher positive schizotypy was associated with enhanced memory specificity in the present study. This finding is in line not only with previous findings, but it may also highlight a core, unifying and more central feature of schizophrenia. Bleuler [24] conceptualized schizophrenia as “a mind that is torn asunder” by loosening associations [cited by 56]. He suggested that the fragmentation of thought, emotion and volition should be viewed as the core psychological feature of the disorder [29]. While the inherently subjective nature of fragmentation renders it challenging to measure, it manifests itself in pervasive disturbances in mental processes, some of which are observable and quantifiable. Evidence suggests that individuals with schizophrenia often struggle with multisensory integration [57, 58]. More importantly, difficulties in information integration within a single cognitive domain such as memory have also been reported. During visual memory tests, patients exhibited a detail-oriented style of processing and were less likely to integrate the details into global features [30, 31]. These results correspond to the theory that there is a local, as opposed to global, processing bias in schizophrenia [28]. Further, Ferman et al. [27] used the global–local paradigm in relation to the different dimensions of the disorder. Importantly, they found that in patients exhibiting positive symptoms, there was a propensity towards local processing which, in turn, interfered with global processing. They argued that this provided evidence for heightened distractibility to feature detail in association with positive symptoms of schizophrenia [27].

Our finding of amplified specificity in high positive schizotypy contrasts with previous studies in patients with schizophrenia which reported a bias towards reduced memory specificity [19–21]. We suggest that this discrepancy might be resolved by considering the heterogeneity of schizophrenia: a bias towards reduced memory specificity might be a marker of accelerated brain aging in schizophrenia [59, 60]. This would be in line with the well-established finding of a positive correlation between a bias towards reduced memory specificity/lure discrimination/pattern separation and age [48, 49, 61, 62] [14, for overviews, see e.g., 32]. On the other hand, the positive symptom dimension might be related to increased memory specificity.

Overall, disruption in the integration of information and, in particular, shift towards detail-oriented processing is evident from studies. Our results support some of the earliest phenomenological descriptions of schizophrenia in the literature [24] and are also in line with studies investigating memory alterations in the disorder in that we have found memory specificity to be enhanced in people with higher positive schizotypy.

### Strengths and limitations

The strengths of the study include the comparison of two well-articulated competing hypotheses, the evaluation of alternative explanations through a series of control analyses, and the reliable measurement of psychopathology and memory alterations. One may argue that our study is limited by the sample size. However, it should be noted that to achieve higher statistical power, we increased variability in the sample by oversampling for positive schizotypy, and we also established the high reliability of our key measurements. Furthermore, the suboptimal reliability of the negative and impulsive schizotypy subscale scores, and of the false lure recognition score from the perceptual discrimination task could have biased the estimated effect sizes downwards, limiting the conclusions that can be drawn regarding these constructs. Future studies should use more reliable measurements to evaluate the association of negative and impulsive schizotypy and perceptual discrimination performance with mnemonic discrimination and generalization.

## Conclusion

Deficits in memory, perception, attention, language production, or motor control have all been detected in schizophrenia and, with lower effect sizes, in people showing high levels of schizotypal traits [1, 2]. Memory dysfunction is considered to be a particularly pronounced symptom of schizophrenia [63], as well as being one of the strongest predictors of illness outcome [64, 65], however, opposing hypotheses emerged in the literature regarding whether patients are more biased toward overgeneralization, or detail-oriented memories. Over and above contrasting the two frameworks, our aim was to investigate whether people with higher positive schizotypal traits in the general population would also show a specific bias in memory. Our results suggest that people who are prone to odd, delusion-like beliefs, and unusual, hallucination-like experiences are also more likely to struggle to get hold of the global picture. The fragmentation of memory representations has an important implication on how we interpret the world around ourselves and how we synthesize information. Thus, people with higher positive schizotypal traits may be more susceptible to experience unusual perceptions and come to erroneous conclusions as they may be unable to look beyond the details and take the broad picture into account. Future high-powered studies with patients should examine the relationship between variation in symptoms and behavioral indicators of pattern separation and completion.

## Supplementary Information

Below is the link to the electronic supplementary material.

Supplementary file1 (PDF 125 KB)

## Footnotes

1 We note that individuals who were invited to participate in the study as part of the oversampling completed the short O-LIFE questionnaire again during the lab testing session, as it was not possible to connect their scores from the oversampling form with their lab test data. Some participants in the high schizotypy subsample obtained less than 7 points on the Unusual Experiences subscale—such a change in observed scores is in line with the test-retest reliability of the subscale [35–37], Still, the mean score of the Unusual Experiences subscale was remarkably high in this group, as compared to the convenience sample.
