# Supplementary material for: Positive schizotypy is associated with amplified mnemonic discrimination and attenuated generalization
Source: Eur Arch Psychiatry Clin Neurosci. 2022 May 27;273(2):447–58. doi: 10.1007/s00406-022-01430-8 (PMC10070292; doi:10.1007/s00406-022-01430-8)
Supplement: Supplementary file 1 — Supplementary file1 (PDF 125 KB) [file 406_2022_1430_MOESM1_ESM.pdf]

Supplementary Materials

Positive schizotypy is associated with amplified mnemonic discrimination and attenuated generalization

European Archives of Psychiatry and Clinical Neuroscience

Ágota Vass[a], Melinda Becske[b], Ágnes Szőllősi[a,c], Mihály Racsmány[a,c], Bertalan Polner[*a]

[a] Department of Cognitive Science, Budapest University of Technology and Economics, Budapest, Hungary

[b] Department of Psychiatry and Psychotherapy, Semmelweis University, Budapest, Hungary

[c] Institute of Cognitive Neuroscience and Psychology, Research Centre for Natural Sciences, Budapest, Hungary

[*] Correspondence concerning this article should be addressed to Bertalan Polner at polner.bertalan@ttk.hu. (Postal address: 1111-Budapest, Hungary, Egry József utca 1; telephone: +3614631273; fax: +3614631273)

Supplementary Materials

Methods

The Oxford-Liverpool Inventory of Feelings and Experiences (O-LIFE) Questionnaire

Participants completed the short version of the O-LIFE questionnaire which measures schizotypal personality traits. It consisted of 43 yes/no questions [1] [Hungarian version 2]. The questionnaire has four subscales: Unusual Experiences (i.e., positive schizotypy) which yielded a satisfactory internal reliability of a Cronbach's alpha (internal consistency reliability in the sample: $\alpha = 0.8$, 12 items); Cognitive Disorganisation (internal consistency reliability in the sample: $\alpha = 0.7$, 11 items); Introvertive Anhedonia (i.e., negative schizotypy) (internal consistency reliability in the sample: $\alpha = 0.4$, 10 items); and Impulsive Nonconformity (internal consistency reliability in the sample: $\alpha = 0.4$, 10 items). The items of this questionnaire are deliberately framed in a less clinical way to make it suitable for tapping schizotypal personality traits in healthy individuals [3].

Questionnaires Assessing Further Trait- and State-Like Psychopathology

As sleep disturbances have been shown to be correlated with schizotypy[4], participants completed the Athen Insomnia Scale (AIS) [5] [Hungarian version: , 6] which is designed for quantifying sleep difficulty based on the ICD-10 criteria (internal consistency reliability in the sample: $\alpha = 0.8$, 8 items). Participants also filled out the General Health Questionnaire-12 (GHQ-12) [7] [Hungarian version 8] which is a well-established screening instrument for symptoms of anxiety and depression, in addition to being a more general psychological/mental well-being assessment (internal consistency reliability in the sample: $\alpha = 0.8$, 12 items). Participants completed the Current Psychotic-like Experiences (internal consistency reliability in the sample: $\alpha = 0.7$) 9-item test [9]. The Hungarian version of this test was created by merging three independent translations, and discrepancies were discussed until an agreement was reached. Finally, participants also filled out the State-Trait Anxiety Inventory (STAI-S) [10] [Hungarian version 11] which measured the anxiety levels of participants (internal consistency reliability in the sample: $\alpha = 0.9$, 20 items). The STAI

includes 20 items on the current level of anxiety. Participants were asked to rate the items of the questionnaire on a 4-point scale.

Mnemonic Similarity Task

The Mnemonic Similarity Task (MST) was developed to assess memory specificity and generalization corresponding to pattern separation and pattern completion on a behaviour level, respectively [12, 13]. The MST is a modified object recognition test that is widely used to assess hippocampal function integrity [13]. It has proved to be useful in detecting hippocampal-based memory alterations across the lifespan [14] and it may also reveal impairments associated with clinical pathology in a variety of disorders, including depression [15], schizophrenia [16–18], and Alzheimer's disease [19].

The stimulus set consisted of colourful photographs of everyday objects presented on a white background (for stimulus exemplars, see Figure 1). The task comprised two phases with no delay between them. In the incidental encoding phase, participants were asked to judge the indoor/outdoor nature of objects via response buttons (F and K on a standard keyboard of the computer, respectively). There were no pre-established right or wrong answers. Each picture was shown for 2 s (with an inter-stimulus interval [ISI] of 0.5 s).

Participants were not informed that the second phase, which followed the encoding phase immediately, would consist of a recognition memory test. In the recognition memory task, three types of pictures were shown on screen: exact repetitions of objects shown in the previous phase (targets), new objects (foils) and perceptually similar items (lures). Participants were asked to identify them correctly as "old" "new" or "similar" via button press (F, K, and H, respectively) (for more details, see Figure 1). Each stimulus was presented for 2 s (ISI = 0.5 s). Correct similar responses to lure items presumably require distinct representations of partially overlapping features, consistent with the neurocomputational model of pattern separation [13]. It should be noted that while pattern separation is the underlying neural computation, lure discrimination is the behavioural manifestation of it, so the term LDI (Lure Discrimination Index) will be used here when referring to memory performance [13, 20]. Behavioural manifestation of pattern completion, on the other hand, is defined as falsely

identifying a lure as an old item because the common qualities of the encoded and lure items presumably represent partial cues from which the encoded item is incorrectly retrieved [21]. We will refer to the behavioural manifestation of this computation as “false recognition of lures”.

The LDI and false recognition of lures were calculated for each participant based on their performance in the second phase. The false recognition of lures was calculated as the difference between the rate of “old” responses given to lure items minus “old” responses given to the foil items to account for any response bias to use the “old” response overall during the test. The LDI was, on the other hand, calculated as the difference between the rate of “similar” responses given to the lure items minus “similar” responses given to the foil items to account for any response bias to use the “similar” response overall during the test. Reliability of the LDI and false recognition of lures in the sample have been evaluated with the Spearman-Brown reliability estimate with split-half correlations using 5000 random samples (taking trial type into account). These analyses indicated that reliability in the sample was good for both the false recognition of lures (mean split-half $r = 0.78$ [2.5% quantile: 0.69; 97.5% quantile: 0.84]) and the LDI (mean split-half $r = 0.80$ [2.5% quantile: 0.73; 97.5% quantile: 0.86]).

Perceptual Discrimination Task

To control for perceptual deficits, participants took part in a Perceptual Discrimination (PD) test immediately after the completion of the MST. In the PD test, participants judged whether 90 pairs of images were identical to, different from, or similar to each other. Stimuli were colourful images of objects and there was no overlap between the stimulus set of the PD task and the MST. Each trial consisted of the subsequent presentations of two images (1 s/first stimulus; 2 s/second stimulus, ISI = 0.5 s). Participants were asked to identify them correctly as “old”, “new”, or “similar” via button press (F, K, and H, respectively). Similar to the MST, false recognition of lures (difference between the rate of “old” responses given to lure items minus “old” responses given to the foil items) and LDI (difference between the rate of “similar” responses given to the lure items minus “similar” responses given to the foil items) for each participant were calculated based on their ability to discriminate between the images. Reliability of the PD LDI and false recognition of lures in the sample have been

evaluated with the Spearman-Brown reliability estimate with split-half correlations using 5000 random samples (taking trial type into account). These analyses indicated that reliability in the sample was good-excellent for the PD LDI (mean split-half r = 0.85 [2.5% quantile: 0.78; 97.5% quantile: 0.90]), however, the PD false lure recognition score had poor reliability (mean split-half r = 0.45 [2.5% quantile: 0.21; 97.5% quantile: 0.65]).

Supplementary Figure 1. The distribution of variables measured in a variety of questionnaires used to assess individual differences in schizotypal traits, general mental health, anxiety, and insomnia.

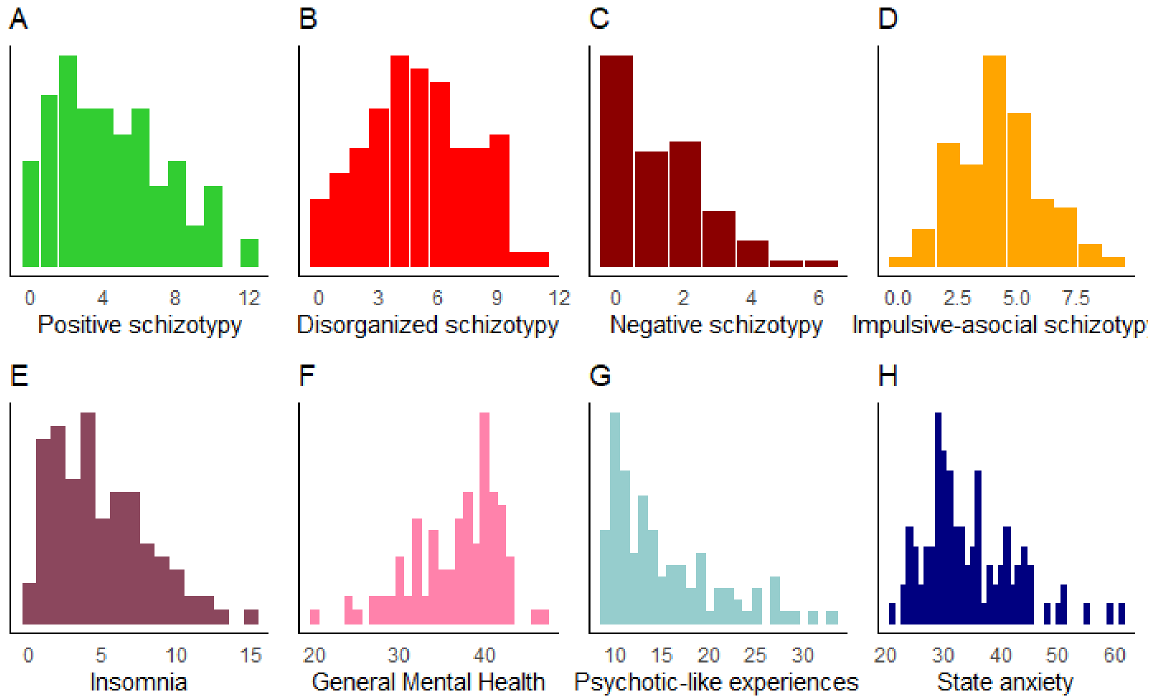

References

1. Mason OJ, Linney Y, Claridge G (2005) Short scales for measuring schizotypy. Schizophrenia Research 78:293–296. https://doi.org/10.1016/j.schres.2005.06.020
2. Kéri S (2011) Solitary minds and social capital: Latent inhibition, general intellectual functions and social network size predict creative achievements. Psychology of Aesthetics, Creativity, and the Arts 5:215–221. https://doi.org/10.1037/a0022000
3. Mason OJ (2015) The Assessment of Schizotypy and Its Clinical Relevance. Schizophr Bull 41:S374–S385. https://doi.org/10.1093/schbul/sbu194
4. Koffel E, Watson D (2009) Unusual sleep experiences, dissociation, and schizotypy: Evidence for a common domain. Clinical Psychology Review 29:548–559. https://doi.org/10.1016/j.cpr.2009.06.004
5. Soldatos CR, Dikeos DG, Paparrigopoulos TJ (2000) Athens Insomnia Scale: validation of an instrument based on ICD-10 criteria. Journal of Psychosomatic Research 48:555–560. https://doi.org/10.1016/S0022-3999(00)00095-7
6. Novak M (2004) Sleep disorders and quality of life. Doctoral dissertation. PhD Diss, Semmelweis Egyetem
7. Goldberg DP, Gater R, Sartorius N, et al (1997) The validity of two versions of the GHQ in the WHO study of mental illness in general health care. Psychological Medicine 27:191–197
8. Balajti I, Vokó Z, Ádány R, Kósa K (2007) Validation of the Hungarian versions of the abbreviated sense of coherence (SOC) scale and the general health questionnaire (GHQ-12). Mentálhigiéné és Pszichoszomatika 8:147–161. https://doi.org/10.1556/Mental.8.2007.2.4
9. Cristóbal-Narváez P, Sheinbaum T, Myin-Germeys I, et al (2017) The role of stress-regulation genes in moderating the association of stress and daily-life psychotic experiences. Acta Psychiatrica Scandinavica 136:389–399. https://doi.org/10.1111/acps.12789
10. Spielberger CD, Gorsuch RL, Lushene R, et al (1983) Manual for the State-Trait Anxiety Inventory. Consulting Psychologists Press, Palo Alto, CA
11. Sipos K, Sipos M, Spielberger CD (1988) A State-Trait Anxiety Inventory (STAI) magyar változata. In: Mérei F, Szakács F (eds) Pszichodiagnosztikai Vademecum I/2. Tankönyvkiadó, Budapest, pp 123–136
12. Stark SM, Yassa MA, Lacy JW, Stark CEL (2013) A task to assess behavioral pattern separation (BPS) in humans: Data from healthy aging and mild cognitive impairment. Neuropsychologia 51:2442–2449. https://doi.org/10.1016/j.neuropsychologia.2012.12.014
13. Stark SM, Kirwan CB, Stark CEL (2019) Mnemonic Similarity Task: A Tool for Assessing Hippocampal Integrity. Trends in Cognitive Sciences 23:938–951. https://doi.org/10.1016/j.tics.2019.08.003
14. Stark SM, Stevenson R, Wu C, et al (2015) Stability of age-related deficits in the mnemonic similarity task across task variations. Behavioral Neuroscience 129:257–268. https://doi.org/10.1037/bne0000055
15. Camfield DA, Fontana R, Wesnes KA, et al (2018) Effects of aging and depression on mnemonic discrimination ability. Aging, Neuropsychology, and Cognition 25:464–483. https://doi.org/10.1080/13825585.2017.1325827
16. Das T, Ivleva EI, Wagner AD, et al (2014) Loss of pattern separation performance in schizophrenia suggests dentate gyrus dysfunction. Schizophrenia Research 159:193–197. https://doi.org/10.1016/j.schres.2014.05.006
17. Martinelli C, Shergill SS (2015) Clarifying the role of pattern separation in schizophrenia: The role of recognition and visual discrimination deficits. Schizophrenia Research 166:328–333. https://doi.org/10.1016/j.schres.2015.06.004
18. Kraguljac NV, Carle M, Frölich MA, et al (2018) Mnemonic Discrimination Deficits in First-Episode Psychosis and a Ketamine Model Suggests Dentate Gyrus Pathology Linked to N-Methyl-D-Aspartate Receptor Hypofunction. Biological Psychiatry: Cognitive Neuroscience and Neuroimaging 3:231–238. https://doi.org/10.1016/j.bpsc.2017.02.005
19. Webb CE, Foster CM, Horn MM, et al (2020) Beta-amyloid burden predicts poorer mnemonic discrimination in cognitively normal older adults. NeuroImage 221:117199. https://doi.org/10.1016/j.neuroimage.2020.117199

20. Szőllősi Á, Racsmány M (2020) Enhanced mnemonic discrimination for emotional memories: the role of arousal in interference resolution. Mem Cogn 48:1032–1045. https://doi.org/10.3758/s13421-020-01035-3
21. Molitor RJ, Ko PC, Hussey EP, Ally BA (2014) Memory-related eye movements challenge behavioral measures of pattern completion and pattern separation. Hippocampus 24:666–672. https://doi.org/10.1002/hipo.22256
